# Systematic engineering to enhance valencene production in *Rhodobacter sphaeroides*

**DOI:** 10.1186/s40643-025-00942-0

**Published:** 2025-09-20

**Authors:** Zhizhen Li, Wenhao Li, Xinyu Gao, Wenming Yao, Zhenqian Zhu, Xueyi Luo, Yang Zhang, Jifeng Yuan

**Affiliations:** State Key Laboratory of Cellular Stress Biology, School of Life Sciences, Faculty of Medicine and Life Sciences, Xiamen University, Fujian, 361102 China

**Keywords:** Rhodobacter sphaeroides, Valencene, Metabolic engineering, Process optimization, Agricultural waste

## Abstract

Microbial synthesis of (+)-valencene from agricultural wastes such as cornstalk will serve as a sustainable alternative to the traditional plant extraction method. In this study, *Rhodobacter sphaeroides* strains were engineered for valencene production through a stepwise manner: (1) heterologous expression of *Callitropsis nootkatensis* valencene synthase (CnVS) in combination with *phaB/gdhA/ladH* knockouts enabled *de novo* biosynthesis of valencene from glucose at a titer of 34.21 ± 3.1 mg/L; (2) a quorum-sensing promoter P_*cer*_ to decouple growth and production phase further improved the valencene titer to 80.75 ± 3.0 mg/L; and (3) transposon-mediated genomic integration of the heterologous mevalonate pathway to enhance farnesyl pyrophosphate supply resulted in 120.53 ± 10.34 mg/L valencene. Subsequently, the alkali-pretreated cornstalk hydrolysate was used as the substrate, and 100.51 ± 14.15 mg/L valencene was achieved under the optimized carbon-to-nitrogen ratio. In summary, the engineered *R. sphaeroides* offers an alternative mean to valorize the cheap agricultural waste for high-value valencene production.

**Supplementary Information:**

The online version contains supplementary material available at 10.1186/s40643-025-00942-0.

## Introduction

(+)-Valencene is a volatile sesquiterpenoid compound characterized by its pleasant citrus-like aroma, which is also known as Valencia orange terpene or Velenciene (Elston et al. 2005). It serves as a food flavor enhancer and a cosmetic fragrance ingredient with extensive applications, and its global annual demand exceeds 10,000 kg (Beekwilder et al. 2014; Hu et al. 2024). Valencene can also be readily converted to (+)-nootkatone by chemical (Wang et al. 2015) or biological means (Liu et al. 2022), which is not only a commonly used flavor compound in soft drinks but also can be used as mosquito repellant (Zhang et al. 2023). Current valencene supply primarily relies on extraction from natural sources (e.g., citrus fruits) or chemical synthesis (Coates and Shaw 1970). However, plant extraction is susceptible to climatic and seasonal variations, and suffers from low valencene yields (0.2%-0.6%) when using grapefruit peel as the feedstock (Beekwilder et al. 2014). Chemical synthesis entails high environmental pollution and significant energy consumption, and faces a challenge in separating chiral subtypes due to the complex structure of sesquiterpenes (Mai et al. 2021).

With the rapid advancement of metabolic engineering, heterologous biosynthesis of terpenoids such as valencene in microbial cell factories represents a promising alternative strategy to plant extraction and chemical synthesis. In recent years, *Escherichia coli*, *Saccharomyces cerevisiae*, and *Komagataella phaffii* (*Pichia pastoris*) have been employed as the microbial chassis for valencene production from glucose (Cao et al. 2022; Chen et al. 2022; Cheng et al. 2025). For instance, Cheng et al. enhanced the native mevalonate pathway, down-regulated the competitive pathway, and overexpressed the fusion enzyme of farnesyl diphosphate synthase (FPP) and valencene synthase (VS) to achieve 173.6 mg/L valencene in shake flasks using *K. phaffii* (Cheng et al. 2025). Chen et al. utilized the protein engineering to improve the native mevalonate pathway and enhance the catalytic activity of valencene synthase, reaching 813 mg/L valencene by *Yarrowia lipolytica* in shake flasks (Chen et al. 2025). Cao et al. compared four types of isoprenoid pathways in the peroxisome of *S. cerevisiae* for valencene production, and eventually employed the optimally *Haloarchaea*-type mevalonate pathway to produce 110.6 mg/L valencene in shake flasks (Cao et al. 2023).Ye et al. engineered *S. cerevisiae* by combining gene screening, protein engineering, and biosynthetic pathway optimization to achieve 16.6 g/L valencene from glucose under the fed-batch fermentation in 15 L bioreactors, which represents the highest valencene titer reported to date (Ye et al. 2022). Metabolic engineers also attempted to directly produce valencene from CO_2_ using cyanobacteria. For instance, Dietsch et al. constructed a fusion protein of FPP synthase and VS, achieving a final yield of 19 mg/g in engineered *Synechocystis sp.* PCC 6803 (Dietsch et al. 2021), which is relatively low for practical applications.

Considering agricultural wastes such as cornstalk are cheap feedstocks that contain abundant fermentable sugars, it will be desirable to take advantage of agricultural wastes for a more sustainable biomanufacturing process (Aggarwal et al. 2024). However, the above-mentioned microbial strains are not capable to naturally utilize the xylose fraction from lignocellulose hydrolysates. And the organic acids from lignocellulose hydrolysates such as formate and acetate would inhibit the cell growth of many microorganisms. In comparison, *Rhodobacter sphaeroides* is a metabolically versatile microorganism that is capable of utilizing diverse carbon sources including agricultural residues (e.g., xylose, arabinose, glucose) and industrial wastewater components (e.g., formate, acetate) for bioproduction applications (Orsi et al. 2021; Zhang et al. 2020; Zhou et al. 2014). Specially, *R. sphaeroides* harnesses the unique ethylmalonyl-CoA (EMC) pathway for acetyl-CoA assimilation instead of the defective glyoxylate shunt (Filatova et al. 2005), which endows preponderant carbon efficiency due to the transformation of 3 mol acetyl-CoA into 2 mol malate along with fixing 2 mol CO_2_ compared to the glyoxylate shunt with requiring 4 mol acetyl-CoA (Alber 2010). Moreover, among gram-negative bacteria, *R. sphaeroides* is regarded as a nonpathogenic bacterium owing to the entirely nontoxic lipopolysaccharide for humans and animals (Wada et al. 2022). And its endogenous 2-C-methyl-D-erythritol-4-phosphate (MEP) pathway supplies isopentenyl diphosphate (IPP) and dimethylallyl diphosphate (DMAPP) for carotenoid synthesis (Chi et al. 2015; Qiang et al. 2019), making it an excellent host for valencene biosynthesis from agricultural wastes.

**Fig. 1:**
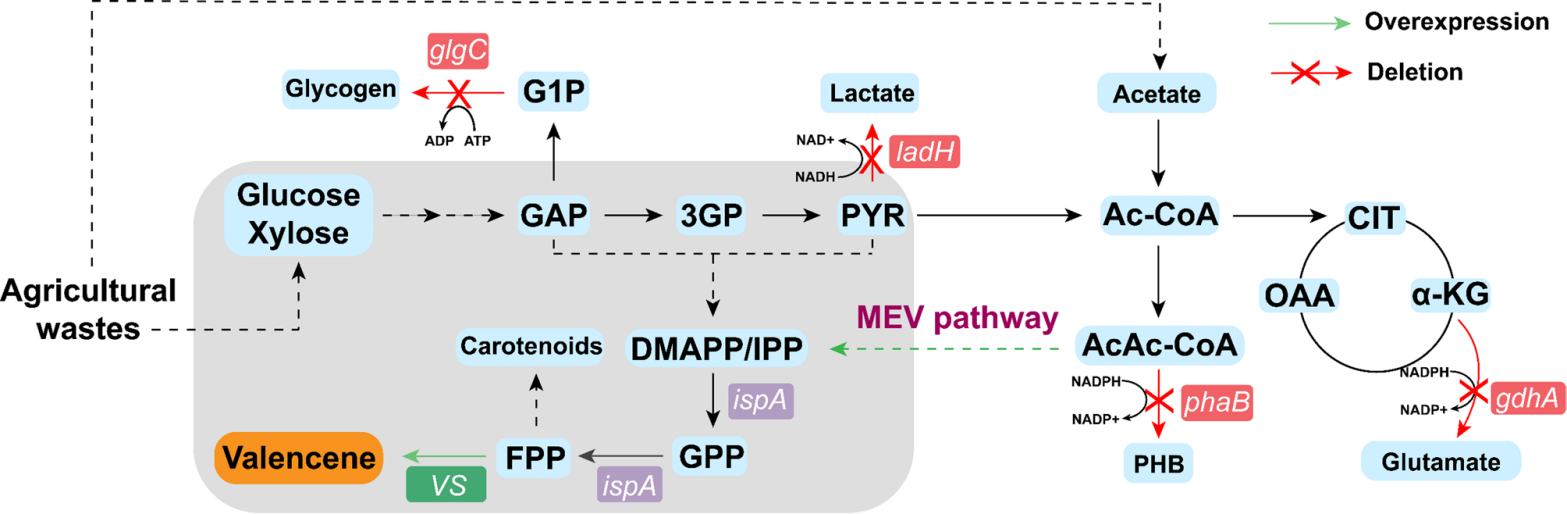
The biosynthesis of valencene in *R. sphaeroides*. G1P: glucose-1-phosphate, GAP: glyceraldehyde-3-phosphate, 3GP: 3-phosphoglycerate, PYR: pyruvate, Ac-CoA: acetyl-coenzyme A, CIT: citrate, OAA: oxaloacetate, α-KG: α-ketoglutarate, AcAc-CoA: acetoacetyl-coenzyme A, PHB: polyhydroxybutyrate, DMAPP/IPP: dimethylallyl pyrophosphate/isopentenyl pyrophosphate, GPP: geranyl pyrophosphate, FPP: farnesyl pyrophosphate, VS: valencene synthase, IspA: FPP synthase, *glgC*: glucose-1-phosphate adenylyltransferase gene, *ladH*: lactate dehydrogenase gene, *phaB*: β-ketoacyl-ACP reductase gene, *gdhA*: glutamate dehydrogenase gene

In this study, we sought to develop *R. sphaeroides* for valencene production from cornstalk hydrolysate. Systematic optimization strategy was implemented to improve the valencene production in engineered *R. sphaeroides* (Fig. 1). First, valencene synthases from different sources were compared and the valencene synthase from *Callitropsis nootkatensis* (CnVS) was identified with a better valencene titer of 5.79 ± 0.41 mg/L. Subsequent knockout of NADPH consumption and byproduct-related genes such as ∆*phaB/gdhA/ladH* gave an elevated titer by 5.9-fold to 34.21 ± 3.1 mg/L. A quorum-sensing promoter P_*cer*_ to decouple growth and production phase further improved the valencene titer to 80.75 ± 3.0 mg/L. Transposon-mediated genomic integration of the heterologous mevalonate (MEV) pathway to enhance FPP supply eventually resulted in 120.53 ± 10.34 mg/L valencene. Finally, the alkali-pretreated cornstalk hydrolysate was employed as the substrate, and 100.51 ± 14.15 mg/L (+)-valencene in shake-flasks was achieved via an optimized fermentation process.

## Materials and methods

### Strains and culture cultivation

*R. sphaeroides* 2.4.1 (Kontur et al. 2012) was used as the parental strain for all genetic manipulations, and its derivatives were aerobically cultivated at 35℃ in Sistrom’s mineral medium (MedA) (Kovach et al. 1995) supplemented with antibiotics (10 mg/L kanamycin, 12 mg/L gentamycin) if needed. *E. coli* TOP10 used for general plasmid constructions and *E. coli* S17-1 used for conjugation mating, were incubated at 37℃ in Luria-Bertani (LB) medium (yeast extract 5 g/L, tryptone 10 g/L, NaCl 10 g/L) added antibiotics (50 mg/L kanamycin, 12 mg/L gentamycin) when necessary. All strains used in this study are listed in Table S1.

### Construction of plasmids

All primers used in this study are listed in Table S2 and synthesized from Sangon Biotech (Shanghai, China). High-Fidelity Phusion DNA polymerase employed for PCR, restriction endonucleases, and T4 DNA ligase were purchased from New England Biolab (Ipswich, MA, USA). RaPure DNA Clean Up Kit and RaPure Plasmid Micro Kit were obtained from Magen Biotech (Guangzhou, China). 2X M5 HiPer plus Taq HiFi PCR Mix used for colony PCR was supplied by Mei5bio (Beijing, China). Detailed descriptions on plasmids construction are presented in Supplementary Information, and all plasmids used in this study are listed in Table S3.

### Di-parental conjugation

Plasmids were transformed into *R. sphaeroides* through conjugation mating. Briefly, the donor of *E. coli* S17-1 carrying the target plasmid and the receptor of *R. sphaeroides* were grown to the mid-log phase, and then collected, washed once with MedA and resuspended with MedA of 1 mL, respectively. *R. sphaeroides* and *E. coli* were mixed at a ratio of 10:3 (v/v) for conjugation on a MedA plate and cultivated overnight at 35℃. Then, the mixture was harvested, washed once with MedA, and spread on a MedA plate with appropriate antibiotics. After incubation at 35℃ for 2–3 days, colonies were chosen for following experiments.

### Screening of Recombinant strains

Homologous recombination mediated by a suicide plasmid pK18mobsacB, which harbors the lethal gene *sacB* to sucrose for selecting double crossover, was utilized for genome editing in *R. sphaeroides*. After conjugation mating, a few colonies containing the pK18mobsacB-derived plasmid were incubated overnight in MedA of 1 mL at 35℃, and then the culture was diluted, spread on MedA plates supplemented with 8% sucrose, and incubated at 30℃ for 2–3 days. Positive colonies were further confirmed by diagnostic PCR and DNA sequencing. Transposon mutagenesis was conducted by the plasmid pRL27 (Larsen et al. 2002) bearing the mini-Tn5 transposon elements with a kanamycin resistance. After conjugation mating, cultures were diluted, spread on MedA plates supplemented with kanamycin, and cultivated at 30℃ for 2–3 days. Positive colonies were further confirmed by kanamycin and diagnostic PCR, and chosen as candidates for subsequent studies.

### Pretreatment of cornstalk

Dried cornstalk was smashed and screened through 80 meshes. The powder samples were mixed with water at a solid-liquid ratio of 1:10 (w/v) and added NaOH to the final dose of 0.75%. The mixture was hydrolyzed at 120℃ for 30 min, and the pH was adjusted to 4.8 using 5 M H_2_SO_4_. Then, cellulase (ACMEC, Shanghai, China) of 0.1 mg/g and hemicellulose (ACMEC, Shanghai, China) of 0.1 mg/g were added to the mixture to react at 50℃ for 10 h. Finally, the cornstalk hydrolysate was obtained by vacuum filtration and its pH was adjusted to 7.0 for microbial fermentation. The composition of the cornstalk hydrolysate was analyzed by a high-performance liquid chromatography (HPLC, Shimadzu LC-20 A, Japan) equipped with a refractive index detector and an Aminex HPX-87 H column (Bio-Rad, CA, USA). The column temperature was set at 50 °C and 5 mM sulfuric acid was used as the mobile phase with a flow rate of 0.5 mL/min.

### Two-phase fermentation for valencene production

For batch fermentation, *R. sphaeroides* strains were pre-cultivated in MedA at 35℃ for 24 h, and then the cultures were incubated in a modified MedA medium (20 g/L glucose or the cornstalk hydrolysate as the carbon source instead of succinate and 7.5 g/L yeast extract as the nitrogen source instead of ammonium sulfate) at 30℃ and 250 rpm. Shake-flask experiments were carried out in triplicate containing 20 mL fermentation media, 1% inoculum, and 20% (v/v) *n*-dodecane, and screening experiments were performed in culture tubes containing 2 mL fermentation media, 1% inoculum, and 20% (v/v) *n*-dodecane. The C/N ratio in the fermentation was controlled by adjusting the doses of cornstalk hydrolysate and yeast extract. The carbon in yeast extract was ignored because its concentration was extremely low compared to that of the hydrolysate (Ma et al. 2022), and the total nitrogen in yeast extract was 10.9%. The C/N ratio was generated using the following formula 1, where *G*, *X*, *R*, *F*, *A*, *Y* represent the doses of glucose, xylose, arabinose, formate, acetate, and yeast extract, respectively.

$$\begin{array}{l}\\\:\frac{Carbon\:of\:cornstalk}{Nitrogen\:of\:yeast\:extract}=\\\frac{\frac{G}{180.16\:g/mol}\times\:6+\:\frac{X+R}{150.13\:g/mol}\times\:5+\frac{F}{46.03\:g/mol}+\frac{A}{60.05\:g/mol}\times\:2}{\frac{10.9\%\times\:Y}{14g/mol}}\:\end{array}$$

### Extraction and detection of products

The upper organic phase was collected and filtered through a 0.22 μm organic nylon membrane. Then, the samples were detected with a gas chromatograph (Shimadzu GC-2030, Japan) equipped with a flame ionization detector and the SH-Rtx-5 capillary column (30 m×0.25 mm×0.25 μm). Nitrogen was employed as a carrier with a flow rate of 1.0 mL/min. The inlet temperature was 250℃, and the injection volume was 1 µL. The column temperature was maintained at 80℃ for 2 min, then raised to 200℃ using a rate of 5℃/min and raised to 300℃ using a rate of 20℃/min. Caryophyllene was utilized as the external standard for drawing the standard curve. The statistical significance of data (*p* value) was analyzed with GraphPad Prism 8 using a *t* test.

## Results and discussion

### Construction of a valencene biosynthesis pathway in R. sphaeroides

**Fig. 2:**
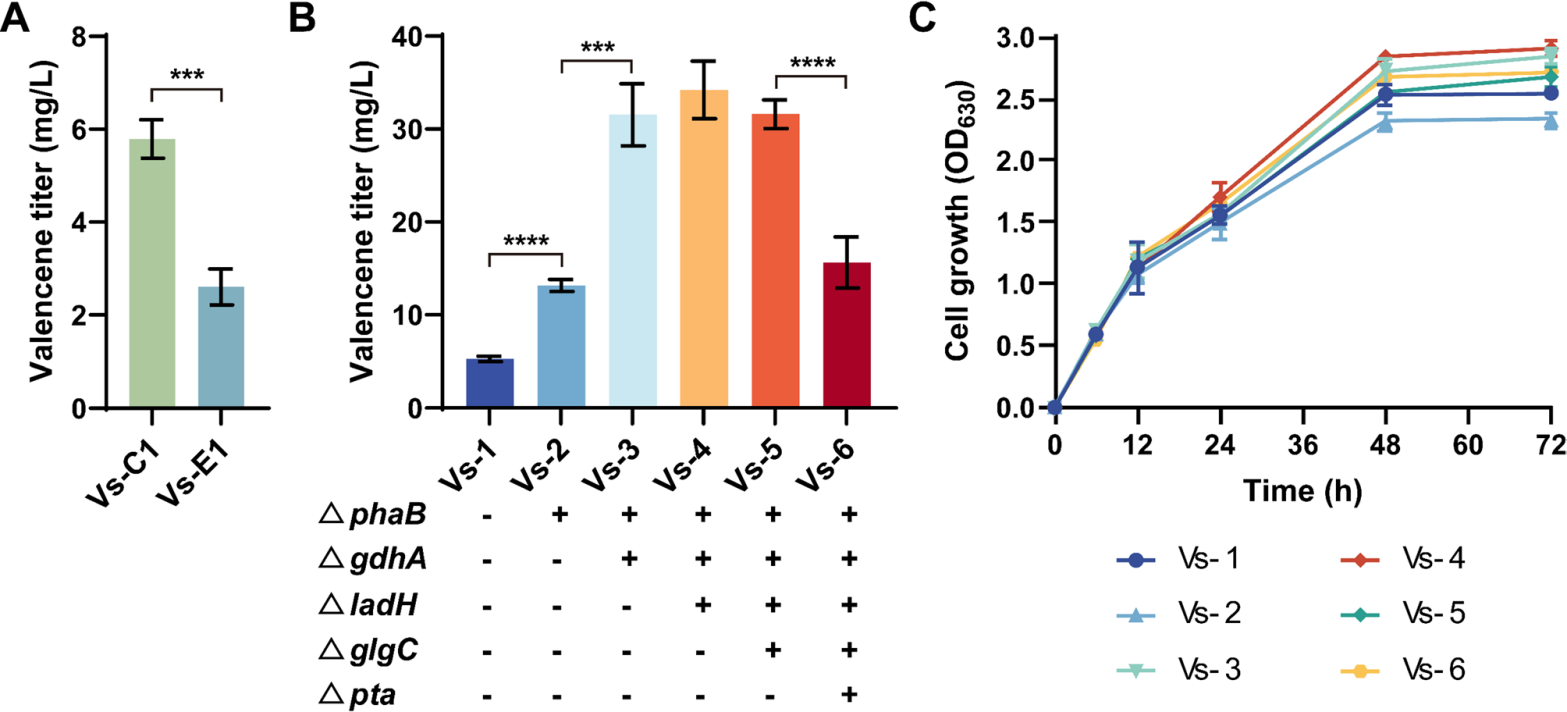
Engineering *R. sphaeroides* for enhanced valencene biosynthesis via metabolic pathway optimization. (**A**) Valencene production in strains expressing CnVS (Vs-C1) and EgVS (Vs-E1) after 72 h of fermentation. (**B**) The valencene production levels differed among mutant strains after 72 h of growth. (**C**) The growth curves of valencene-producing strains. All experiments were carried out with three independent biological replicates. Data represent the average ± SD from the triplicate experiments. Three asterisks indicate statistically significant results *p* < 0.001 and four asterisks indicate *p* < 0.0001

As the wild-type *R. sphaeroides* lacks valencene synthase, it necessitates the introduction of heterologous enzymes for valencene production. To establish valencene biosynthesis as depicted in Fig. 1, valencene can be synthesized from FPP derived from the MEP pathway. The activities of valencene synthases from *C. nootkatensis* (CnVS) (Beekwilder et al. 2014) and *Eryngium glaciale* (EgVS) (Ye et al. 2022) under the control of constitutive promoter P_*tac*_ were compared in *R. sphaeroides*. It was found that the heterologous expression of CnVS in *R. sphaeroides* resulted in a higher valencene titer than that of EgVS (Fig. 2A). Therefore, CnVS from *C. nootkatensis* was used for the subsequent valencene production in *R. sphaeroides*.

As the MEP pathway for synthesizing 1 mol valencene requires 8 mol NADPH, we attempted to improve the valencene production by enhancing NADPH supply as depicted in Fig. 1. Since the polyhydroxybutyrate (PHB) biosynthesis is an NADPH consuming pathway, the PHB synthesis pathway was further engineered to restrict carbon flux toward this pathway, thereby enhancing NADPH accumulation (Mougiakos et al. 2019). As shown in Fig. 2B, knockout of NADPH-consuming *phaB* gene involved in PHB synthesis resulted in a 127% increase over the control strain Vs-1 (viz. Vs-C1), reaching 13.15 ± 0.65 mg/L valencene. Subsequently, another NADPH-consuming glutamate dehydrogenase encoded by *gdhA* gene was disrupted as it was previously reported to increase terpenoid synthesis in *Rhodobacter capsulatus* (Zhang et al. 2021). The resulting ∆*phaB*∆*gdhA* double-knockout strain (Vs-3) led to a further elevated valencene titer of 31.53 ± 3.35 mg/L (Fig. 2B). The shake-flask fermentation results showed that deletion of these genes had negligible effects on cell growth (Fig. 2C), whereas the growth of mutant strain Vs-3 was substantially improved than that the parental strain Vs-1.

As minimizing fermentation byproduct formation represents an effective strategy for biochemical productions (Lai et al. 2022; Mo et al. 2022; Yang et al. 2022), next a set of byproduct-related genes were knocked out as described in Fig. 1. Given that lactate dehydrogenase (encoded by the *ladH* gene) also consumes carbon flux and reducing power of NADH, the *ladH* gene was further deleted in the Vs-3 background. This triple-knockout strain (Vs-4: ∆*phaB/gdhA/ladH*) reached a valencene yield of 34.21 ± 3.1 mg/L and the cell growth of Vs-4 was also improved over that of Vs-3 (Fig. 2C). Next, the *glgC* gene encoding glucose-1-phosphate adenylyltransferase was knocked out in the Vs-4 background, aiming to block the carbon flow towards glycogen storage and redirect the metabolic flux towards targeted valencene synthesis. However, the resulting quadruple-knockout strain (Vs-5: ∆*phaB/gdhA/ladH/glgC*) did not give an expected improvement on valencene production, and a slightly lower titer of 31.61 ± 1.55 mg/L valencene was observed in strain Vs-5, indicating that the metabolic flux of glycogen synthesis might be insignificant. Moreover, strategic deletion of the *pta* gene encoding phosphate acetyltransferase in the Vs-5 background aimed to reduce carbon loss via the acetate pathway. Unexpectedly, the mutant strain (Vs-6: ∆*phaB/gdhA/ladH/glgC/pta*) exhibited a noticeable deleterious effect on valencene synthesis, with the titer reducing to 15.63 ± 2.75 mg/L. Since phosphate acetyltransferase (PTA) primarily participates in acetate metabolism and the synthesis/utilization of acetyl-CoA in microorganisms, and *R. sphaeroides* lacks a typical pyruvate oxidase (POX) (Kontur et al. 2012), it was suggested that PTA might play an indispensable role in acetyl-CoA homeostasis in *R. sphaeroides*. Overall, triple mutation of Δ*phaB*, Δ*gdhA*, and Δ*ladH* could divert more metabolic flux toward valencene biosynthesis, reaching an approximate 6-fold increase (34.21 ± 3.1 mg/L vs. initial 5.79 ± 0.41 mg/L).

### Dynamic regulation of valencene synthase in R. sphaeroides

Precise control of gene expression is a pivotal step in metabolic engineering, where key pathway enzymes can maximize targeted product synthesis (Pérez et al. 2022). Promoters play critical roles in regulating gene expression by primarily controlling transcription initiation (He et al. 2023). Xu et al. systematically optimized the expression of the *Enterobacter cloacae* gene cluster using promoters of varying strengths (P_*T7*_, P_*tac*_, P_*C*_, P_*abc*_) to enhance 2,3-butanediol production while alleviating metabolic burden in *E. coli* (Xu et al. 2014). In addition, tuning promoter strength is essential for optimal heterologous gene expression (Blazeck and Alper 2013). More recently, quorum sensing (QS)-mediated temporal gene regulation through diffusible autoinducer molecules has been widely implemented for metabolic engineering applications (Parsek and Greenberg 2000; Pollitt et al. 2014; Waters and Bassler 2005). Upon reaching threshold concentrations at a certain cell density, autoinducers bind its cognate receptors to modulate downstream transcription in a cell density dependent manner (Parsek and Greenberg 2000; Pollitt et al. 2014; Waters and Bassler 2005), which results in a balanced cell growth and biochemical production.

**Fig. 3:**
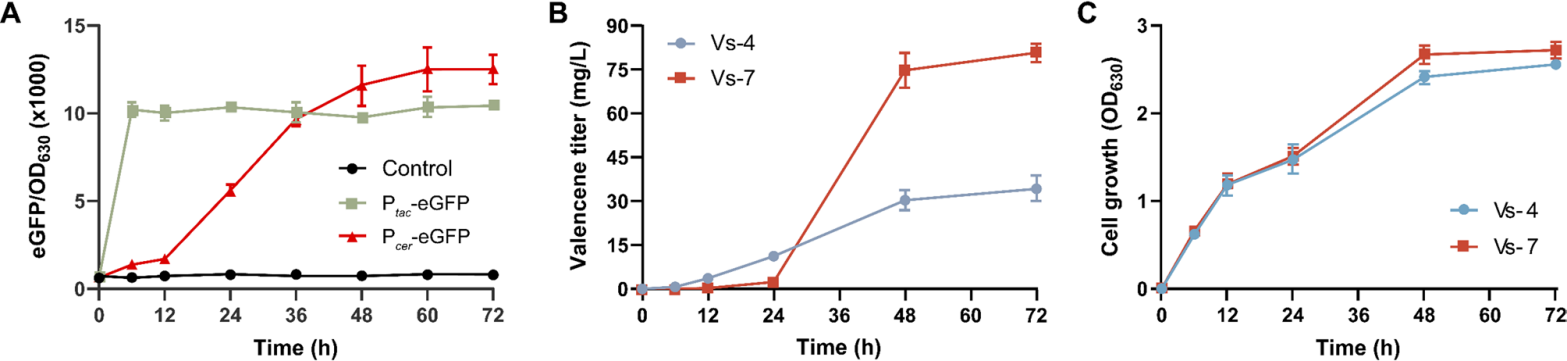
Quorum sensing-mediated optimization of valencene production in engineered *R. sphaeroides*. (**A**) Time course of the expression profiles of the QS system and constitutive promoter using an eGFP-reporter. (**B**) Valencene production in engineered *R. sphaeroides* Vs4 (*CnVS* driven by P_*tac*_ with the deletion of *phaB*, *gdhA*, and *ladH*) and Vs-7 (*CnVS* driven by P_*cer*_-mediated QS regulation with the deletion of *phaB*, *gdhA*, and *ladH*). (**C**) Growth curve of the valencene-producing strain under P_*cer*_-mediated QS regulation. All experiments were carried out with three independent biological replicates. Data represent the average ± SD from the triplicate experiments

In *R. sphaeroides*, the core QS component *cerI* synthase catalyzes the formation of the specific autoinducer 7,8-*cis*-*N*-(tetradecenoyl)homoserine lactone (C_14_-HSL) (Puskas et al. 1997). At a certain cell density, C_14_-HSL binds the receptor CerR, forming a complex that activates the downstream P_*cer*_ promoter to drive high-level expression of targeted genes. In order to compare the QS promoter P_*cer*_ and the constitutive promoter P_*tac*_, the expression dynamics were characterized using enhanced green fluorescent protein (eGFP) as a reporter. As shown in Fig. 3A, compared to constitutive P_*tac*_ promoter-driven eGFP expression, the QS system exhibited a distinct population density-dependent dynamic. Notably, the QS system induced a more robust activation of eGFP expression relative to the constitutive P_*tac*_ promoter. These findings confirmed that the QS promoter P_*cer*_ could turn on the targeted gene expression in response to the cell population density.

To further decouple the growth and production phase for improving valencene biosynthesis, the QS promoter P_*cer*_ was implemented to control CnVS expression. As shown in Fig. 3B and C, the valencene production was extremely low in the engineered strain Vs-7 with P_*cer*_-*CnVS* during the early stage of cell growth (0–24 h), which was obviously less than the engineered strain Vs-4 with P_*tac*_-*CnVS*. Nevertheless, the valencene titer of strain Vs-7 substantially increased to 80.75 ± 3.0 mg/L during the mid to late stage of cell growth (24–72 h), eventually representing a 236.04% increase over that of strain Vs-4. Moreover, it was noticed that a comparatively positive effect on the cell density using the QS circuit, as the biomass accumulation was improved during late fermentation process. Therefore, successful implementation of the population-dependent expression of *CnVS* mediated by the P_*cer*_ promoter demonstrated a beneficial role to achieve a balanced cell growth and valencene production, confirming the robust applicability of the QS system as a dynamic regulatory tool in synthetic biology for constructing high-performance microbial cell factories.

### Enhancing valencene biosynthesis through genomic integration of the MEV pathway

**Fig. 4:**
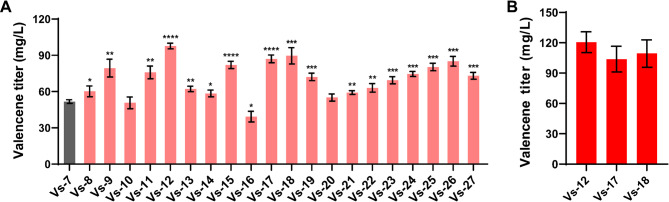
Enhancing valencene precursor supply through heterologous terpenoid pathway introduction. (**A**) Screening of high-yield valencene mutant strains via transposon-integrated MEV pathway in culture tubes. (**B**) Shake-flask fermentation of high-yield valencene mutants. All experiments were carried out with three independent biological replicates. Data represent the average ± SD from the triplicate experiments. When comparing the valencene titer of Vs-7, one asterisk indicates statistically significant results *p* < 0.05, two asterisks indicate *p* < 0.005, three asterisks indicate *p* < 0.001, and four asterisks indicate *p* < 0.0001

Although enhancements in isoprenoid biosynthesis were reached by optimizing the MEP pathway, this strategy most likely is subject to restrictions because of the control mechanisms present in the native host such as the feedback inhibition of 1-deoxy-D-xylulose-5-phosphate synthase and 2 C-methyl-D-erythritol-2,4-cyclo-diphosphate synthase of the MEP pathway (Li et al. 2020). To bypass restrictions of the MEP pathway that may beattributed to the unknown physiological regulatory elements in *R. sphaeroides*, a heterologous MEV pathway from *Paracoccus zeaxanthinifaciens* was introduced into strain Vs-7 to enhance the FPP supply to achieve a further improved valencene productivity. For stable genomic integration, transposon-mediated mutagenesis was employed: the pRL27 plasmid, carrying Tn5 transposase, facilitates random insertion of the MEV gene cluster into the host genome, to yield a mutant library with distinct performances. Upon conjugation transferring the pRL-MEV plasmid (Fig. S1) into the recipient host Vs-7, a number of mutants were screened for valencene production. Fermentations in tubes revealed 85% (17/20) of isolates surpassed the valencene titer of the parental triple-knockout strain Vs-7 (51.74 ± 1.53 mg/L) (Fig. 4A). Among them, three high-yield strains were successfully validated through shake-flask fermentations, with the mutant Vs-12 exhibiting the highest valencene titer of 120.53 ± 10.34 mg/L (Fig. 4B). The results indicated that co-expression of the native MEP and exogenous MEV pathways, which substantially enhanced the biosynthesis of IPP and DMAPP, thereby channeling more carbon flux preferentially towards FPP formation (Schempp et al. 2017; Zhang et al. 2021), in engineered *R. sphaeroides* led to a considerable increase in valencene production. It was reported that four types of isoprenoid biosynthetic pathways (the classical MEV, *Haloarchaea*-type MEV, *Thermoplasma*-type MEV, and isoprenoid alcohol pathways) were applied to evaluate valencene production in *S. cerevisiae*, and their impacts on valencene synthesis displayed distinct differences, in which the *Haloarchaea*-type MEV pathway operated the optimal effect (Cao et al. 2023). Hence, given the classical MEV pathway was harnessed in this work, other types of isoprenoid biosynthetic pathways are worthy of being further explored in *R. sphaeroides*. In addition, the results also confirmed that transposon mutagenesis is an effective method to generate a combinatorial genome integration library with distinct biochemical productivities, which is likely to rewrite the global cellular metabolism and optimize cell robustness through blocking, down-regulating, or up-regulating gene expressions. However, deciphering of underneath mechanism for the improved valencene titer in certain mutants might require technologies like genome sequencing, omics and bioinformatics.

Compared to the microbial fermentation of valencene previously reported, the valencene production of this work has the considerable room for improvement. For instance, FPP is not only the direct precursor to valencene biosynthesis, but also used for ubiquinone, cell wall, and pigments biosynthesis in *R. sphaeroides*; therefore, the balance between FPP to valencene biosynthesis and the competed pathways should be explored in the future through replacing with weaken promoters of key genes (Cheng et al. 2025), or CRISPR interference on key enzymes (Zhang et al. 2021). Since VS is a pivotal enzyme for valencene biosynthesis, efficient approaches including the fusion enzyme of FPP synthase and VS to spatial colocalization of enzymes for facilitating cascade catalysis (Chen et al. 2025), the integration of proper multi-copies of VS into the genome instead of plasmid expression (Cao et al. 2023), and the protein engineering of predicted enzyme reaction cavity by site-directed mutagenesis based on the multiple sequence alignment result of the target enzyme and its orthologues (Chen et al. 2025; Ye et al. 2022) or the information of protein crystal structures, could be appropriate for improving valencene production in *R. sphaeroides*. Of note, the scalable valencene bioproduction needs the synergistic combination of genetic modification and fermentation optimization (Song et al. 2024), and medium components, fermentation parameters, and bioreactor configurations are critical for the optimal engineered strain.

### Direct production of valencene from agricultural waste

**Fig. 5:**
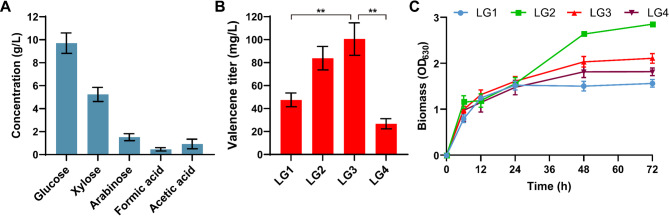
*De novo* synthesis of valencene from cornstalk. (**A**) Composition analysis of cornstalk hydrolysate following combined alkaline-enzymatic pretreatment (**B**) The influence of varying C/N ratios on valencene biosynthesis. (**C**) The growth curves of valencene-producing strains under different C/N ratio fermentation conditions. LG1: low C/N (7:1), LG2: moderate C/N (10:1), LG3: moderate C/N (17:1); LG4: high C/N (34:1). All experiments were carried out with three independent biological replicates. Data represent the average ± SD from the triplicate experiments. Two asterisks indicate statistically significant results *p* < 0.005

Cornstalk consists primarily of cellulose, hemicellulose, and lignin, with the native lignocellulosic recalcitrance limiting sugar release (Hendriks and Zeeman 2009). Pretreatment is therefore essential to disrupt the lignin barrier and enhance subsequent saccharification efficiency (Wyman et al. 2005). Cornstalk powder was pretreated and diluted to yield glucose (9.71 ± 0.89 g/L), xylose (5.24 ± 0.62 g/L), arabinose (1.52 ± 0.3 g/L) and other organic acids (Fig. 5A). The short-chain organic acids are assimilated via the native EMC pathway in *R. sphaeroides*, replenishing tricarboxylic acid (TCA) cycle intermediates to provide essential energy and biosynthetic precursors, while contributing to suppressing contaminant growth (Shimizu et al. 2019). All of these carbon sources are key energy and carbon substrates that support *R. sphaeroides* growth and metabolism.

To establish efficient valencene biosynthesis from cornstalk hydrolysate, fermentation conditions were optimized. In this study, we attempted to investigate the temporal profiles of biomass accumulation and valencene synthesis across varying C/N ratios to identify the optimal valencene production conditions using the strain Vs-12. As shown in Fig. 5B, high C/N (34:1) resulted in a minimum valencene titer (26.75 ± 4.39 mg/L), whereas low C/N (7:1) enhanced biomass formation (Fig. 5C) but only achieved 47.55 ± 5.96 mg/L valencene. At the balanced C/N ratio of 17:1, carbon and nitrogen availability reached an optimal metabolic state to support an optimal valencene production of 100.51 ± 14.15 mg/L at 72 h (Fig. 5B). These findings indicated that varying C/N ratios is critical for an optimal valencene production from cornstalk hydrolysate. Therefore, our study demonstrated that hydrolytically pretreated agricultural wastes could be directly utilized by *R. sphaeroides* as carbon sources, achieving biosynthesis efficiency comparable to premium carbon substrates such as glucose. In future study, beyond strain productivity optimization, fermentation optimization in large scale bioreactors must be considered (Song et al. 2024) such as the parameter optimization including dissolved oxygen, maintenance of pH homeostasis and feeding strategy, the rich media optimization instead of minimal media, and the extraction agent optimization of two-phase fermentation for overproduction and product separation.

## Conclusion

In summary, our work achieved improved valencene biosynthesis in *R. sphaeroides* while exploring sustainable production using renewable resources. Through systematic metabolic engineering strategies including heterologous pathway construction, byproduct and cofactor engineering, dynamic pathway regulation, and precursor supply optimization, the valencene production was substantially enhanced in *R. sphaeroides*. Cornstalk hydrolysate was examined for valencene production, reaching 100.51 ± 14.15 mg/L in small scale shake-flasks. Despite the valencene titers remain below industrial requirements, continued efforts such as the protein engineering of CnVS and fermentation optimization in large scale bioreactors will eventually achieve economical valencene biosynthesis from agricultural wastes.

## Supplementary Information

Below is the link to the electronic supplementary material.

Supplementary Material 1: This file contains Supplementary Materials and Methods, gene sequences, plasmid map of pRL-MEV (Fig. S1), list of strains used in this study (Table S1), list of primers used in this study (Table S2), and list of plasmids used in this study (Table S3)

Supplementary Material 2

## Data Availability

Not applicable.

## Abbreviations

CnVS: Valencene synthase from Callitropsis nootkatensis
C_14_-HSL: 7,8-cis-N-(tetradecenoyl)homoserine lactone
DMAPP: Dimethylallyl diphosphate
eGFP: Enhanced green fluorescent protein
EgVS: Valencene synthase from Eryngium glaciale
EMC: Ethylmalonyl-CoA
FPP: Farnesyl pyrophosphate
IPP: Isopentenyl diphosphate
IspA: Farnesyl diphosphate synthase
MEP: 2-C-methyl-D-erythritol-4-phosphate
MEV: Mevalonate
PHB: Polyhydroxybutyrate
PTA: Phosphate acetyltransferase
QS: Quorum sensing
TCA: Tricarboxylic acid
VS: Valencene synthase
