# Supplementary material for: Systematic engineering to enhance valencene production in Rhodobacter sphaeroides
Source: Bioresour Bioprocess. 2025 Sep 20;12(1):100. doi: 10.1186/s40643-025-00942-0 (PMC12449282; doi:10.1186/s40643-025-00942-0)
Supplement: Supplementary file 1 — Supplementary Material 1: This file contains Supplementary Materials and Methods, gene sequences, plasmid map of pRL-MEV (Fig. S1), list of strains used in this study (Table S1), list of primers used in this study (Table S2), and list of plasmids used in this study (Table S3) [file 40643_2025_942_MOESM1_ESM.docx]

**Supplementary Information**

**Systematic engineering to enhance valencene production in *Rhodobacter sphaeroides***

Zhizhen Li^1,#^, Wenhao Li^1,#^, Xinyu Gao^1,#^, Wenming Yao^1^, Zhenqian Zhu^1^, Xueyi Luo^1^, Yang Zhang^1,^*, Jifeng Yuan^1,^*****

^1^ State Key Laboratory of Cellular Stress Biology, School of Life Sciences, Faculty of Medicine and Life Sciences, Xiamen University, Fujian 361102, China

^#^ These authors contributed equally.

* Corresponding authors: [jfyuan@xmu.edu.cn](mailto:jfyuan@xmu.edu.cn) (J.Y.) or [y-zhang@xmu.edu.cn](mailto:y-zhang@xmu.edu.cn) (Y.Z.)

**Supplementary Materials and Methods**

**Plasmids construction**

The plasmid pARtacT derived from the vector pBBR1MCS-2 (Kovach et al., 1995) was engineered to express genes in *Rhodobacter sphaeroides*. The P*_cer_* promoter amplified from the genome of *R. sphaeroides* with primers Pcer-F/Pcer-R was cloned into pABRtacT digested by *Eco*RI and *Kpn*I to generate pARPcerT. The DNA fragments of CnVS and EgVS were codon-optimized for *R. sphaeroides* and synthesized by Genscript (Nanjing, China), and cloned into pARPcerT via *Bam*HI and *Xho*I to obtain pARPcer-CnVS and pARPcer-EgVS, respectively. To construct pK29-ΔphaB, the upstream and downstream fragments of the *phaB* gene were amplified from *R. sphaeroides* genome with primers phaBup-F/phaBup-OE-R and phaBdow-OE-F/phaBdow-R and assembled by overlapping PCR, then cloned into pK29mobSacB via *Xba*I and *Kpn*I. Using the similar method, the plasmids, pK29-ΔgdhA, pK29-ΔladH, pK29-ΔglgC, and pK29-Δpta, were obtained. The DNA fragment of the mevalonate (MEV) pathway operon from *Paracoccus zeaxanthinifaciens* ATCC 21588 synthesized from GenScript (Nanjing, China) was cloned into pRL27DeBX digested by *Esp*3I to give pRL-MEV.

Valencene synthase gene from *Callitropsis nootkatensis* (*CnVS*)

*GGTCTCGGATCC*ATGGCCGAAATGTTCAATGGCAATTCCAGCAATGATGGCAGCTCCTGCATGCCGGTCAAGGACGCGCTGCGCCGCACCGGGAACCACCATCCGAACCTCTGGACCGACGATTTCATCCAGTCGCTGAACTCCCCCTATTCGGATTCCTCGTATCATAAACATCGCGAGATCCTGATCGATGAGATCCGGGACATGTTCTCCAACGGCGAGGGGGATGAGTTCGGGGTCCTCGAGAACATCTGGTTCGTCGACGTGGTCCAGCGGCTGGGCATCGATCGGCACTTCCAGGAAGAGATCAAGACGGCCCTGGATTATATCTATAAGTTCTGGAACCATGATAGCATCTTCGGCGACCTCAACATGGTGGCGCTGGGGTTCCGCATCCTGCGGCTCAATCGCTACGTGGCGTCGTCGGACGTGTTCAAGAAGTTCAAGGGCGAGGAGGGCCAGTTCTCGGGGTTCGAGAGCAGCGATCAGGACGCCAAGCTGGAGATGATGCTGAACCTCTACAAGGCCTCGGAACTCGACTTCCCGGATGAGGACATCCTCAAGGAAGCGCGGGCCTTCGCGTCGATGTATCTCAAGCATGTCATCAAGGAGTATGGGGACATCCAGGAATCGAAGAACCCCCTGCTCATGGAGATCGAGTACACCTTCAAGTACCCCTGGCGCTGCCGCCTCCCGCGGCTGGAGGCGTGGAACTTCATCCACATCATGCGGCAGCAGGACTGCAATATCTCGCTCGCCAACAACCTCTATAAGATCCCGAAGATCTATATGAAGAAGATCCTGGAGCTGGCGATCCTCGACTTCAACATCCTCCAGAGCCAGCATCAGCATGAGATGAAACTGATCAGCACGTGGTGGAAGAACTCGTCCGCGATCCAGCTCGACTTCTTCCGCCACCGCCATATCGAGAGCTACTTCTGGTGGGCCAGCCCGCTGTTCGAGCCCGAGTTCTCCACCTGCCGCATCAACTGCACCAAGCTGTCCACCAAGATGTTCCTCCTGGACGACATCTATGACACGTACGGGACCGTCGAGGAACTCAAGCCGTTCACGACCACCCTCACGCGCTGGGATGTCAGCACGGTGGACAATCACCCGGACTACATGAAGATCGCGTTCAATTTCTCCTACGAGATCTACAAGGAGATCGCGTCCGAGGCCGAGCGCAAGCACGGCCCGTTCGTGTATAAGTATCTCCAGTCGTGCTGGAAGTCGTATATCGAGGCGTATATGCAGGAGGCCGAGTGGATCGCCTCCAACCACATCCCCGGCTTCGACGAGTACCTGATGAATGGCGTGAAGAGCTCGGGGATGCGCATCCTCATGATCCATGCGCTGATCCTGATGGATACGCCCCTGTCCGACGAGATCCTCGAGCAGCTCGACATCCCGAGCAGCAAGAGCCAGGCCCTGCTGTCGCTCATCACGCGGCTCGTCGATGATGTGAAGGATTTCGAGGACGAGCAGGCGCATGGGGAGATGGCCTCGTCGATCGAATGCTATATGAAGGATAATCACGGCTCCACGCGCGAGGACGCCCTGAACTACCTGAAAATCCGCATCGAGAGCTGCGTGCAGGAGCTCAACAAGGAACTCCTCGAACCGAGCAACATGCATGGCAGCTTCCGCAACCTGTACCTCAACGTGGGCATGCGGGTGATCTTCTTCATGCTGAACGACGGGGACCTCTTCACCCATTCGAATCGGAAGGAGATCCAGGATGCGATCACGAAGTTCTTCGTGGAACCGATCATCCCGTGATAACTCGAGCCAGGCATCAAATAAAACGAAAGGCTCAGTCGAAAGACTGGGCCTTTCGTTTTATCTGTTGTTTGTCGGTGAACGCTCTCTACTAGAGTCACACTGGCTCACCTTCGGGTGGGCCTTTCTGCGTTTATACCTAG*CTCGAGGAGACC*

Valencene synthase gene from *Eryngium glaciale* (*EgVS*)

*GGATCC*ATGAGCCTCAACGTGCTGTCCACGTCGGGCTCGGCGCCCACCACAAAGTCCTCGGAGATCACCCGTCGCAGTGCCAACTATCATCCTAGCCTCTGGGGCGACAAATTTTTAGAATATTCCTCTCCCGACCATTTGAAGAACGACTCGTTCACCGAGAAAAAGCACGAGCAGCTCAAAGAGGAGGTGAAGAAGATGCTGGTCGAGACGGTGCAGAAGCCGCAGCAGCAGCTCAATCTGATCAACGAGATCCAGCGGCTCGGGCTCAGCTACCTCTTCGAGCCGGAGATCGAGGCGGCCCTGCAGGAGATCTCGGTCACCTATGACGAGTTCTGCTGCTCGACCGATGCCGACGATCTTCACAATGTGGCCCTCTCCTTCCGCATTCTGCGGGAGCACGGCCATAACGTCTCCAGCGATGTGTTCCAGAAGTTCATGGATTCGAACGGCAAGCTGAAGGACTATCTGGTGAATGATGCGCGGGGACTACTCAGTCTCTATGAGGCCACCCATTTCCGCGTGCACAACGACGATAAGCTCGAAGAGCTCCTGTCGGTGACGACCTCGCGCCTGGAGCACCTCAAGAGCCATGTGAAATATCCCCTGGAGGACGAGATCTCCCGGGCGCTCAAGCATCCGCTGCACAAGGAGCTGAACCGCCTCGGGGCGCGCTACTACATCTCGATCTACGAGAAGTTCGACTCCCACAACAAGCTGCTCCTGGAGTTCGCCAAGCTCGACTTCAACCGGCTCCAAAAGATGTATCAGCACGAGCTGGCCCATCTGACGCGGTGGTGGAAGGACCTCGACTTCACCAACAAGCTGCCGTTCGCTCGCGACCGGATCGTCGAGGGGTACTTCTGGATTCTGGGCATGTATTTCGAGCCCGAGCGGAAGGACGTGCGCGAATTCCTGAACCGCGTTTTCGCGCTGATCACGGTGGTCGACGACACCTACGATGTCTATGGCACCTTCAAGGAGCTGCTGCTGTTCACCGACGCCATCGAGCGTTGGGGCACGAGCGATCTGGACCAGCTGCCGGGCTACATGCGCATCATCTACCAGGCGCTGATGGACGTCTACAACCAGATGGAGGAGAAGCTCTCGATGAAGGCAGACTGCCCGACGTATCGCCTGGAGTTCGCGATCGAGACCGTCAAGGCGATGTTCCGGTCCTATCTGGAGGAGGCCCGCTGGTCGAAGGAGCATTACATCCCCTCGATGGAAGAATACATGACGGTGGCGCTCGTCTCGGTGGGCTACAAGACCATCCTCACGAATAGCTTCGTGGGGATGGGCGACATCGCCACGCGCGAGGTCTTTGAATGGGTCTTCAACTCGCCCCTTATCATCCGCGCCTCGGACCTGATCGCGCGCCTTGGGGATGACATCGGCGGCCACGAGGAAGAGCAGAAGAAGGGCGACGCGGCGACGGCCATCGAGTGCTACATTAAGGAGAACCATGTGACCAAGCACGAGGCCTATGACGAATTCCAGAAGCAGATCGACAACGCCTGGAAGGATCTGAACAAGGAAGCGCTGCGCCCCTTCCCGGTGCCGATGACCTTCATCACCCGGGTGGTCCATTTCACGAGAGCGATCCACGTGATCTATGCCGATTTCAGCGACGGTTACACGCGGTCCGACAAGGCCATCCGCGGCTATATCACCTCGCTTCTGGTGGACCCGATCCCGCTCTAA*CTCGAG*

Mevalonate (MEV) pathway operon from *Paracoccus zeaxanthinifaciens*

*GGTCTCGGATCC*ATGAAAGACCAGATGATTTCCCATACCCCGGTGCCCACGCAATGGGTCGGCCCGATCCTGTTCCGCGGCCCCGTCGTCGAGGGCCCGATCAGCGCGCCGCTGGCCACCTACGAGACGCCGCTCTGGCCCTCGACCGCGCGGGGGGCAGGGGTTTCCCGGCATTCGGGCGGGATCCAGGTGTCGCTGGTCGACGAACGCATGAGCCGCTCGATCGCGCTGCGGGCGCATGACGGGGCGGCGGCGACCGCCGCCTGGCAGTCGATCAAGGCCCGCCAGGAAGAGGTCGCGGCCGTGGTCGCCACCACCAGCCGCTTCGCCCGCCTTGTCGAGCTGAATCGCCAGATCGTGGGCAACCTGCTTTACATCCGCATCGAATGCGTGACGGGCGACGCCTCGGGTCACAACATGGTCACCAAGGCCGCCGAGGCCGTGCAGGGCTGGATCCTGTCGGAATACCCGATGCTGGCCTATTCCACGATCTCGGGGAACCTGTGCACCGACAAGAAGGCGTCGGCGGTCAACGGCATCCTGGGCCGCGGCAAATACGCCGTCGCCGAGGTCGAGATCCCGCGCAAGATCCTGACCCGCGTGCTGCGCACCAGCGCCGAGAAGATGGTCCGCCTGAACTACGAGAAGAACTATGTCGGGGGTACGCTGGCGGGGTCGCTGCGCAGTGCGAACGCGCATTTCGCCAACATGCTGCTGGGCTTCTACCTGGCGACGGGGCAGGACGCGGCCAACATCATCGAGGCCAGCCAGGGCTTCGTCCATTGCGAGGCCCGCGGCGAGGATCTGTATTTCTCGTGCACGCTGCCCAACCTCATCATGGGCTCGGTCGGTGCCGGCAAGGGCATCCCCTCGATCGAGGAGAACCTGTCGCGGATGGGCTGCCGCCAGCCGGGCGAACCCGGCGACAACGCGCGCCGTCTTGCGGCGATCTGCGCGGGCGTCGTGCTGTGTGGTGAATTGTCGCTGCTTGCGGCCCAGACCAACCCCGGAGAGTTGGTCCGCACCCACATGGAGATGGAGCGATGACCGACAGCAAGGATCACCATGTCGCGGGGCGCAAGCTGGACCATCTGCGTGCATTGGACGACGATGCGGATATCGACCGGGGCGACAGCGGCTTCGACCGCATCGCGCTGACCCATCGCGCCCTGCCCGAGGTGGATTTCGACGCCATCGACACGGCGACCAGCTTCCTGGGCCGTGAACTGTCCTTCCCGCTGCTGATCTCGTCCATGACCGGCGGCACCGGCGAGGAGATCGAGCGCATCAACCGCAACCTGGCCGCTGGTGCCGAGGAGGCCCGCGTCGCCATGGCGGTGGGCTCGCAGCGCGTGATGTTCACCGACCCCTCGGCGCGGGCCAGCTTCGACCTGCGCGCCCATGCGCCCACCGTGCCGCTGCTGGCCAATATCGGCGCGGTGCAGCTGAACATGGGGCTGGGGCTGAAGGAATGCCTGGCCGCGATCGAGGTGCTGCAGGCGGACGGCCTGTATCTGCACCTGAACCCCCTGCAAGAGGCCGTCCAGCCCGAGGGGGATCGCGACTTTGCCGATCTGGGCAGCAAGATCGCGGCCATCGCCCGCGACGTTCCCGTGCCCGTCCTGCTGAAGGAGGTGGGCTGCGGCCTGTCGGCGGCCGATATCGCCATCGGGCTGCGCGCCGGGATCCGGCATTTCGACGTGGCCGGTCGCGGCGGCACATCCTGGAGCCGGATCGAGTATCGCCGCCGCCAGCGGGCCGATGACGACCTGGGCCTGGTCTTCCAGGACTGGGGCCTGCAGACCGTGGACGCCCTGCGCGAGGCGCGGCCCGCGCTTGCGGCCCATGATGGAACCAGCGTGCTGATCGCCAGCGGCGGCATCCGCAACGGTGTCGACATGGCGAAATGCGTCATCCTGGGGGCCGACATGTGCGGGGTCGCCGCGCCCCTGCTGAAAGCGGCCCAAAACTCGCGCGAGGCGGTTGTATCCGCCATCCGGAAACTGCATCTGGAGTTCCGGACAGCCATGTTCCTCCTGGGTTGCGGCACGCTTGCCGACCTGAAGGACAATTCCTCGCTTATCCGTCAATGAAAGTGCCTAAGATGACCGTGACAGGAATCGAAGCGATCAGCTTCTACACCCCCCAGAACTACGTGGGACTGGATATCCTTGCCGCGCATCACGGGATCGACCCCGAGAAGTTCTCGAAGGGGATCGGGCAGGAGAAAATCGCACTGCCCGGCCATGACGAGGATATCGTGACCATGGCCGCCGAGGCCGCGCTGCCGATCATCGAACGCGCGGGCACGCAGGGCATCGACACGGTTCTGTTCGCCACCGAGAGCGGGATCGACCAGTCGAAGGCCGCCGCCATCTATCTGCGCCGCCTGCTGGACCTGTCGCCCAACTGCCGTTGCGTCGAGCTGAAGCAGGCCTGCTATTCCGCGACGGCGGCGCTGCAGATGGCCTGCGCGCATGTCGCCCGCAAGCCCGACCGCAAGGTGCTGGTGATCGCGTCCGATGTCGCGCGCTATGACCGCGAAAGCTCGGGCGAGGCGACGCAGGGTGCGGGCGCCGTCGCCATCCTTGTCAGCGCCGATCCCAAGGTGGCCGAGATCGGCACCGTCTCGGGGCTGTTCACCGAGGATATCATGGATTTCTGGCGGCCGAACCACCGCCGCACGCCCCTGTTCGACGGCAAGGCATCGACGCTGCGCTATCTGAACGCGCTGGTCGAGGCGTGGAACGACTATCGCGCGAATGGCGGCCACGAGTTCGCCGATTTCGCGCATTTCTGCTATCACGTGCCGTTCTCGCGGATGGGCGAGAAGGCGAACAGCCACCTGGCCAAGGCGAACAAGACGCCGGTGGACATGGGGCAGGTGCAGACGGGCCTGATCTACAACCGGCAGGTCGGGAACTGCTATACCGGGTCGATCTACCTGGCATTCGCCTCGCTGCTGGAGAACGCTCAGGAGGACCTGACCGGCGCGCTGGTCGGTCTGTTCAGCTATGGCTCGGGTGCGACGGGC*GAATTC*TTCGATGCGCGGATCGCGCCCGGTTACCGCGACCACCTGTTCGCGGAACGCCATCGCGAATTGCTGCAGGATCGCACGCCCGTCACATATGACGAATACGTTGCCCTGTGGGACGAGATCGACCTGACGCAGGGCGCGCCCGACAAGGCGCGCGGTCGTTTCAGGCTGGCAGGTATCGAGGACGAGAAGCGCATCTATGTCGACCGGCAGGCCTGAAGCAGGCGCCCATGCCCCGGGCAAGCTGATCCTGTCCGGGGAACATTCCGTGCTCTATGGTGCGCCCGCGCTTGCCATGGCCATCGCCCGCTATACCGAGGTGTGGTTCACGCCGCTTGGCATTGGCGAGGGGATACGCACGACATTCGCCAATCTCTCGGGCGGGGCGACCTATTCGCTGAAGCTGCTGTCGGGGTTCAAGTCGCGGCTGGACCGCCGGTTCGAGCAGTTCCTGAACGGCGACCTAAAGGTGCACAAGGTCCTGACCCATCCCGACGATCTGGCGGTCTATGCGCTGGCGTCGCTTCTGCACGACAAGCCGCCGGGGACCGCCGCGATGCCGGGCATCGGCGCGATGCACCACCTGCCGCGACCGGGTGAGCTGGGCAGCCGGACGGAGCTGCCCATCGGCGCGGGCATGGGGTCGTCTGCGGCCATCGTCGCGGCCACCACGGTCCTGTTCGAGACGCTGCTGGACCGGCCCAAGACGCCCGAACAGCGCTTCGACCGCGTCCGCTTCTGCGAGCGGTTGAAGCACGGCAAGGCCGGTCCCATCGACGCGGCCAGCGTCGTGCGCGGCGGGCTTGTCCGCGTGGGCGGGAACGGGCCGGGTTCGATCAGCAGCTTCGATTTGCCCGAGGATCACGACCTTGTCGCGGGACGCGGCTGGTACTGGGTACTGCACGGGCGCCCCGTCAGCGGGACCGGCGAATGCGTCAGCGCGGTCGCGGCGGCGCATGGTCGCGATGCGGCGCTGTGGGACGCCTTCGCAGCCTGCACCCGCGCGTTGGAGGCCGCGCTGCTGTCTGGGGGCAGCCCCGACGCCGCCATCACCGAGAACCAGCGCCTGCTGGAACGCATCGGCGTCGTGCCGGCAGCGACGCAGGCCCTCGTGGCCCAGATCGAGGAGGCGGGTGGCGCGGCCAAGATCTGCGGCGCAGGTTCCGTGCGGGGCGATCACGGCGGGGCGGTCCTCGTGCGGATTGACGACGCGCAGGCGATGGCTTCGGTCATGGCGCGCCATCCCGACCTCGACTGGGCGCCCCTGCGCATGTCGCGCACGGGGGCGGCACCCGGCCCCGCGCCGCGTGCGCAACCGCTGCCGGGGCAGGGCTGATGGATCAGGTCATCCGCGCCAGCGCGCCGGGTTCGGTCATGATCACGGGCGAACATGCCGTGGTCTATGGACACCGCGCCATCGTCGCCGGGATCGAGCAGCGCGCCCATGTGACGATCGTCCCGCGTGCCGACCGCATGTTTCGCATCACCTCGCAGATCGGGGCGCCGCAGCAGGGGTCGCTGGACGATCTGCCTGCGGGCGGGACCTATCGCTTCGTGCTGGCCGCCATCGCGCGACACGCGCCGGACCTGCCTTGCGGGTTCGACATGGACATCACCTCGGGGATCGATCCGAGGCTCGGGCTTGGATCCTCGGCGGCGGTGACGGTCGCCTGCCTCGGCGCGCTGTCGCGGCTGGCGGGGCGGGGGACCGAGGGGCTGCATGACGACGCGCTGCGCATCGTCCGCGCCATCCAGGGCAGGGGCAGCGGGGCCGATCTGGCGGCCAGCCTGCATGGCGGCTTCGTCGCCTATCGCGCGCCCGATGGCGGTGCCGCGCAGATCGAGGCGCTTCCGGTGCCGCCGGGGCCGTTCGGCCTGCGCTATGCGGGCTACAAGACCCCGACAGCCGAGGTGCTGCGCCTTGTGGCCGATCGGATGGCGGGCAACGAGGCCGCTTTCGACGCGCTCTACTCCCGGATGGGCGCAAGCGCAGATGCCGCGATCCGCGCGGCGCAAGGGCTGGACTGGGCTGCATTCCACGACGCGCTGAACGAATACCAGCGCCTGATGGAGCAGCTGGGCGTGTCCGACGACACGCTGGACGCGATCATCCGCGAGGCGCGCGACGCGGGCGCCGCAGTCGCCAAGATCTCCGGCTCGGGGCTGGGGGATTGCGTGCTGGCACTGGGCGACCAGCCCAAGGGTTTCGTGCCCGCAAGCATTGCCGAGAAGGGACTTGTTTTCGATGACTGATGCCGTCCGCGACATGATCGCCCGTGCCATGGCGGGCGCGACCGACATCCGAGCAGCCGAGGCTTATGCGCCCAGCAACATCGCGCTGTCGAAATACTGGGGCAAGCGCGACGCCGCGCGGAACCTTCCGCTGAACAGCTCCGTCTCGATCTCGTTGGCGAACTGGGGCTCTCATACGCGGGTCGAGGGGTCCGGCACGGGCCACGACGAGGTGCATCACAACGGCACGCTGCTGGATCCGGGCGACGCCTTCGCGCGCCGCGCGTTGGCATTCGCTGACCTGTTCCGGGGGGGGAGGCACCTGCCGCTGCGGATCACGACGCAGAACTCGATCCCGACGGCGGCGGGGCTTGCCTCGTCGGCCTCGGGGTTCGCGGCGCTGACCCGTGCGCTGGCGGGGGCGTTCGGGCTGGATCTGGACGACACGGATCTGAGCCGCATCGCCCGGATCGGCAGTGGCAGCGCCGCCCGCTCGATCTGGCACGGCTTCGTCCGCTGGAACCGGGGCGAGGCCGAGGATGGGCATGACAGCCACGGCGTCCCGCTGGACCTGCGCTGGCCCGGCTTCCGCATCGCGATCGTGGCCGTGGACAAGGGGCCCAAGCCTTTCAGTTCGCGCGACGGCATGAACCACACGGTCGAGACCAGCCCGCTGTTCCCGCCCTGGCCTGCGCAGGCGGAAGCGGATTGCCGCGTCATCGAGGATGCGATCGCCGCCCGCGACATGGCCGCCCTGGGTCCGCGGGTCGAGGCGAACGCCCTTGCGATGCACGCCACGATGATGGCCGCGCGCCCGCCGCTCTGCTACCTGACGGGCGGCAGCTGGCAGGTGCTGGAACGCCTGTGGCAGGCCCGCGCGGACGGGCTTGCGGCCTTTGCGACGATGGATGCCGGCCCGAACGTCAAGCTGATCTTCGAGGAAAGCAGCGCCGCCGACGTGCTGTACCTGTTCCCCGACGCCAGCCTGATCGCGCCGTTCGAGGGGCGTTGA*CTCGAGGAGACC*


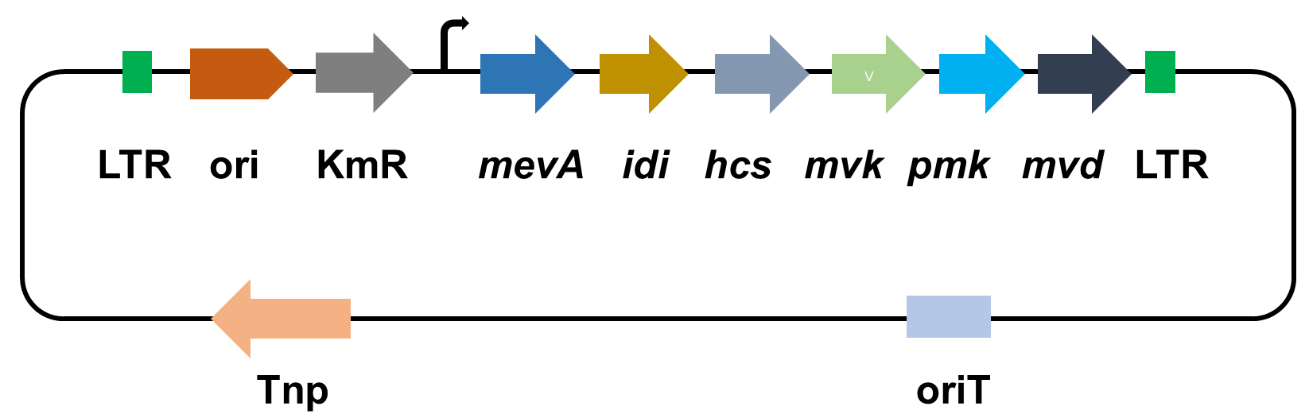


**Fig. S1.** The plasmid map of pRL-MEV. LTR, transposase recognition sites (long terminal repeat); ori, λpir protein-dependent replicator oriR6K; KmR, kanamycin resistance; HMG-CoA, S-3-hydroxy-3-methylglutaryl-CoA; *mevA*, HMG-CoA reductase gene; *idi*, isopentenyl-diphosphate isomerase gene; *hcs*, HMG-CoA synthase gene; *mvk*, mevalonate kinase gene; *pmk*, phosphomevalonate kinase gene; *mvd*, R-5-diphosphomevalonate decarboxylase gene; Tnp, transposase; oriT, origin of transfer.

**Table S1. Strains used in this study**

| **Strains** | **Description** | **Sources** |
| --- | --- | --- |
| *E. coli* S17-1 | *thi pro hsdR hsdM^+^* recA::(RP4-2-Tc::Mu-Km::Tn7) λpir, Sm^r^ | Weidi Biotech |
| *E. coli* Top10 | *F- mcrA Δ(mrr-hsdRMS-mcrBC) φ80 lacZΔM15 ΔlacX74 recA1 araΔ139 Δ(ara-leu)7697 galU galK rpsL (Str^R^) endA1 nupG* | Weidi Biotech |
| *R. sphaeroides* 2.4.1 | Wild type, abbreviated as Rsp2.4.1 | (Kontur et al., 2012) |
| Control | Rsp2.4.1 harboring the empty vector pBRT | This study |
| Vs-C1 | Rsp2.4.1 harboring the plasmid pARPtac-CnVS | This study |
| Vs-E1 | Rsp 2.4.1 harboring the plasmid pARPtac-EgVS | This study |
| Vs-1 | Rsp 2.4.1 harboring the plasmid pARPtac-CnVS | This study |
| Vs-2 | Vs-1 derivative with *phaB* gene deletion | This study |
| Vs-3 | Vs-2 derivative with *gdhA* gene deletion | This study |
| Vs-4 | Vs-3 derivative with *ladH* gene deletion | This study |
| Vs-5 | Vs-4 derivative with *glgC* gene deletion | This study |
| Vs-6 | Vs-5 derivative with *pta* gene deletion | This study |
| Vs-7 | Rsp 2.4.1 derivative with *phaB*, *gdhA* and *ladH* genes deletion harboring the plasmid pARPcer-*CnVS* | This study |
| Vs-8~Vs-27 | Vs-7 with the transposon mutagenesis using pRL-MEV | This study |

**Table S2 Primers used in this study**

| **Name** | **Sequence (5ʹ → 3ʹ)** |
| --- | --- |
| Pcer-F | CGGAATTCAATGGGAGAAGCACAAGC |
| Pcer-R | TTGGTACCTTCTTCAATCCCCAAGGT |
| phaBup-F | GCTCTAGATGCCCTTCGTCATCAAAACCC |
| phaBup-OE-R | TGAGAAGATCCTCAGACGAAACATGATCCCTCCTTCAAATATCCG |
| phaBdow-OE-F | TATTTGAAGGAGGGATCATGTTTCGTCTGAGGATCTTCTCATCC |
| phaBdow-R | TTGGTACCTTGCCAATCCGCCCTACTATC |
| gdhAup-F | TTGGTACCGCGGCGACCTTCAGATTGT |
| gdhAup-OE-R | CTCCTGCCTCAGAGCCTTGCATGTCAGGCCTCCTC |
| gdhAdow-OE-F | GGCCTGACATGCAAGGCTCTGAGGCAGGAGTGGGG |
| gdhAdow-R | GCTCTAGACGGATCGGAAAGATGATACGG |
| glgCup-F | TTGGTACCCAGCCCTGGAAGGCGATGTTG |
| glgCup-OE-R | CTCAGGCTGACCGTCGTGCTGGTGACGCAGGACAT |
| glgCdow-OE-F | CTGCGTCACCAGCACGACGGTCAGCCTGAGTGGGG |
| glgCdow-R | GCTCTAGACTCGGGGATGCGTGAAGC |
| ladHup-F | GCTCTAGACCACGGGAGTTCCAGCAGAT |
| ladHup-OE-R | GGCGCGGCGCCTCAGGCAGGCTCCTTCCGATCATT |
| ladHdow-OE-F | TCGGAAGGAGCCTGCCTGAGGCGCCGCGCCGTTCG |
| ladHdow-R | TTGGTACCACGAGGATGGCGGCGAGCAC |
| ptaup-F | GCTCTAGAAGGGAAAATATGGGCTGGTG |
| ptaup-OE-R | CCACCGGCTTCGTTATGGAAGGATCACATCCGAA |
| ptadow-OE-F | ATGTGATCCTTCCATAACGAAGCCGGTGGCCGAAT |
| ptadow-R | TTGGTACCAGCCCGTGAAAGCCGTAGCG |

**Table S3 Plasmids used in this study**

| **Plasmids** | **Description** | **Sources** |
| --- | --- | --- |
| pBBR1MCS-2 | Broad-host-range vector, Km^r^ | (Kovach et al., 1995) |
| pBRT | pBBR1MCS-2 derivative containing the new MCS and the terminator rrnB T1 | (Wu et al., 2021) |
| pBRPtacT | pBRT derivative carrying the promoter P*_tac_* | Lab storage |
| pABRtacT | pBRPtacT derivative with ampicillin resistance instead of kanamycin resistance, Amp^r^ | Lab storage |
| pRL27 | Transposon plasmid, Km^r^ | (Larsen et al., 2002) |
| pRL27DeBX | pRL27 derivative with the removal of an endogenous *Esp*3I site, and the introduction of the promoter P*_tac_* and two *Esp*3I sites (cohesive ends of *Bam*HI and *Xho*I) | Lab storage |
| pK18mobSacB | Mobilizable suicide vector, *sacB* (sucrose sensitivity), Km^r^ | Invitrogen |
| pK29mobSacB | pK18mobsacB derivative with the removal of two *Kpn*I sites | Lab storage |
| pARPcerT | pABRtacT derivative with the promoter P*_cer_* instead of P*_tac_* | This study |
| pARPtac-CnVS | pABRtacT derivative carrying the *CnVS* driven by P*tac* | This study |
| pARPtac-EgVS | pABRtacT derivative carrying the *EgVS* driven by P*tac* | This study |
| pARPcer-CnVS | pARPcerT derivative carrying the *CnVS* driven by P*cer* | This study |
| pK29-△phaB | pK29mobSacB derivative containing the flanking regions of *phaB* | This study |
| pK29-△gdhA | pK29mobSacB derivative containing the flanking regions of *gdhA* | This study |
| pK29-△ladH | pK29mobSacB derivative containing the flanking regions of *ladH* | This study |
| pK29-△glgC | pK29mobSacB derivative containing the flanking regions of *glgC* | This study |
| pK29-△pta | pK29mobSacB derivative containing the flanking regions of *pta* | This study |
| pRL-MEV | pRL27DeBX derivative carrying the cassette of MEV pathway operon from *P. zeaxanthinifaciens* | This study |

**References**

Kontur WS, Schackwitz WS, Ivanova N et al (2012) Revised sequence and annotation of the *Rhodobacter sphaeroides* 2.4.1 genome. J Bacteriol 194:7016-7017.

Kovach ME, Elzer PH, Hill DS et al (1995) Four new derivatives of the broad-host-range cloning vector pBBR1MCS, carrying different antibiotic-resistance cassettes. Gene 166:175-176.

Larsen RA, Wilson MM, Guss AM, Metcalf WW (2002) Genetic analysis of pigment biosynthesis in *Xanthobacter autotrophicus* Py2 using a new, highly efficient transposon mutagenesis system that is functional in a wide variety of bacteria. Arch Microbiol 178:193-201.

Wu X, Ma G, Liu C, Qiu XY, Min L, Kuang J, Zhu L (2021) Biosynthesis of pinene in purple non-sulfur photosynthetic bacteria. Microb Cell Fact 20:101.
