# Supplementary figures and images for: Systematic engineering to enhance valencene production in Rhodobacter sphaeroides
Source: Bioresour Bioprocess. 2025 Sep 20;12(1):100. doi: 10.1186/s40643-025-00942-0 (PMC12449282; doi:10.1186/s40643-025-00942-0)

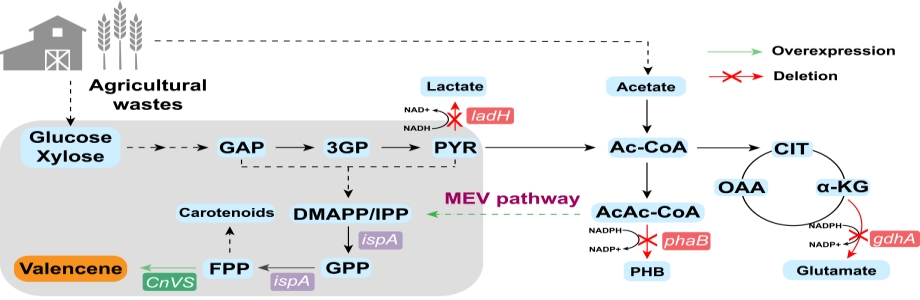

Supplement: Supplementary file 2 — Supplementary Material 2 [file 40643_2025_942_MOESM2_ESM.jpg]
